# Discriminative feature learning for multiclass lung disease classification using contrastive learning

**DOI:** 10.3389/fradi.2026.1824991

**Published:** 2026-08-19

**Authors:** Hafiza Akter Munira, Xuan Zhang, Prathish K. Rajaraman, Alejandro P. Comellas, Eric A. Hoffman, Sean B. Fain, Jiwoong Choi, Mario Castro, Mark L. Schiebler, Elliot Israel, Serpil C. Erzurum, Tianbao Yang, Ching-Long Lin

**Affiliations:** 1 IIHR-Hydroscience & Engineering, University of Iowa, Iowa City, IA, United States; 2 Department of Mechanical Engineering, University of Iowa, Iowa City, IA, United States; 3 Department of Internal Medicine, University of Iowa, Iowa City, IA, United States; 4 Department of Radiology, University of Iowa, Iowa City, IA, United States; 5 Roy J. Carver Department of Biomedical Engineering, University of Iowa, Iowa City, IA, United States; 6 Department of Electrical and Computer Engineering, University of Iowa, Iowa City, IA, United States; 7 Department of Health and Human Physiology, University of Iowa, Iowa City, IA, United States; 8 Division of Pulmonary, Critical Care and Sleep Medicine, University of Kansas School of Medicine, Kansas City, KS, United States; 9 Bioengineering Program, University of Kansas, Lawrence, KS, United States; 10 School of Medicine & Public Health, University of Wisconsin, Madison, WI, United States; 11 Brigham & Women's Hospital, Harvard Medical School, Boston, MA, United States; 12 Cleveland Clinic, Cleveland, OH, United States; 13 Computer Science & Engineering, Texas A&M University, College Station, TX, United States

**Keywords:** asthma, chronic respiratory diseases, computed tomography, contrastive learning, COPD, discriminative embeddings, post-COVID-19

## Abstract

**Objectives:**

To develop a contrastive learning model for lung disease classification using discriminative CT imaging embeddings.

**Methods:**

A total of 1,187 subjects were included: asthma (*n* = 315), COPD (*n* = 355), post-COVID-19 (*n* = 375), and healthy controls (*n* = 142). Of these, 1,003 subjects had a single visit with similarly protocoled CT scans acquired at total lung capacity (TLC) and residual volume (RV), and 92 (33 asthma and 59 post-COVID-19) completed a follow-up visit, with two scans per visit. We developed a modified contrastive learning model incorporating an expert-conditioned routing network and adaptive temperature scaling to learn discriminative embeddings. Model performance was evaluated using the area under the receiver operating characteristic curve (AUC). The embeddings were further validated via k-means clustering, and quantitative CT (qCT) metrics were compared across the derived clusters. The embedding space was used to track disease progression or improvement in the follow-up disease subgroup and to evaluate the model's ability to predict qCT metrics, quantified by the coefficient of determination $\left(R^{2}\right)$.

**Results:**

The model achieved a macro-AUC of 89.3% (95% CI: 86.5, 91.8; *P* < 0.001) in differentiating the four classes. Post-COVID-19 emerged as a distinct class from asthma and COPD in the t-SNE embedding space, and its embeddings across two visits captured disease improvement. Additionally, the learned embeddings showed predictive power for several qCT metrics, particularly the Jacobian ($R^{2}$=0.61).

**Conclusions:**

The proposed model effectively differentiated these three lung diseases and provided meaningful embeddings for phenotype characterization, longitudinal assessment, and qCT metric prediction.

## 1 Introduction

Respiratory diseases pose significant global health challenges, often presenting with overlapping symptoms that complicate diagnosis (1, 2). Among these, post-COVID-19 respiratory sequelae have emerged as a major new health burden (3), alongside established conditions such as Chronic Obstructive Pulmonary Disease (COPD), the fourth leading cause of death globally (4), and asthma, which affects an estimated 262 million people worldwide (5). Accurate differentiation between these diseases is critical for effective treatment; however, traditional diagnostic methods such as spirometry, conventional imaging, and clinical assessment are time-consuming and subject to variability due to patient-specific factors and examiner-dependent interpretation (6, 7). While deep learning has shown promise in medical image analysis (8–10), most existing models focus on binary classification or single-disease detection, leaving a gap in multiclass differentiation and biomarker discovery (11, 12).

Contrastive learning, a self-supervised approach, has emerged as a powerful tool for learning discriminative features from limited labeled data (13, 14). It works by maximizing agreement between augmented views of the same sample while pushing apart dissimilar samples, thereby enhancing model generalizability—a key advantage in medical applications where datasets are often small and heterogeneous (15, 16). However, its potential for multiclass respiratory disease classification and identification of specific imaging metrics remains largely unexplored. While recent research has applied contrastive learning to mitigate demographic biases (17) and established initial strategies for anatomy-specific representation (18), our current work extends these paradigms to specifically minimize disease bias, ensuring the model captures disease specific patterns.

Chest computed tomography (CT) scans provide detailed morphological information essential for assessing patients with chronic respiratory diseases (19–22). Quantitative CT (qCT) enhances these assessments by offering objective metrics beyond conventional CT. In this study, we used similarly protocolled CT scans as the primary imaging modality and generated two-dimensional (2D) projections from three-dimensional (3D) data to enable efficient contrastive learning, with the potential to transfer the model to chest x-rays (CXR). While qCT metrics require costly feature engineering limited to known phenotypes, deep learning can directly learn hierarchical image representations to identify discriminative embeddings. We proposed an expert-conditioned contrastive learning framework to address multiclass respiratory disease classification and to explore correlations between embeddings and qCT metrics. Within this framework, we also incorporated adaptive temperature scaling and lung volume transformation using CT scans acquired at two lung volumes (11).

We hypothesized that our framework could effectively differentiate asthma, COPD, post-COVID-19, and healthy controls. Our aim was to develop a contrastive learning-based model capable of robustly classifying these three lung diseases through discriminative embeddings that capture disease-specific patterns, thereby supporting clinical decision-making and personalized treatment. Furthermore, we evaluated the model's ability to track disease progression or improvement in the patient subgroup and assessed the embeddings' utility for predicting key qCT metrics.

## 2 Materials and methods

### 2.1 Human subject datasets

**Figure 1:**
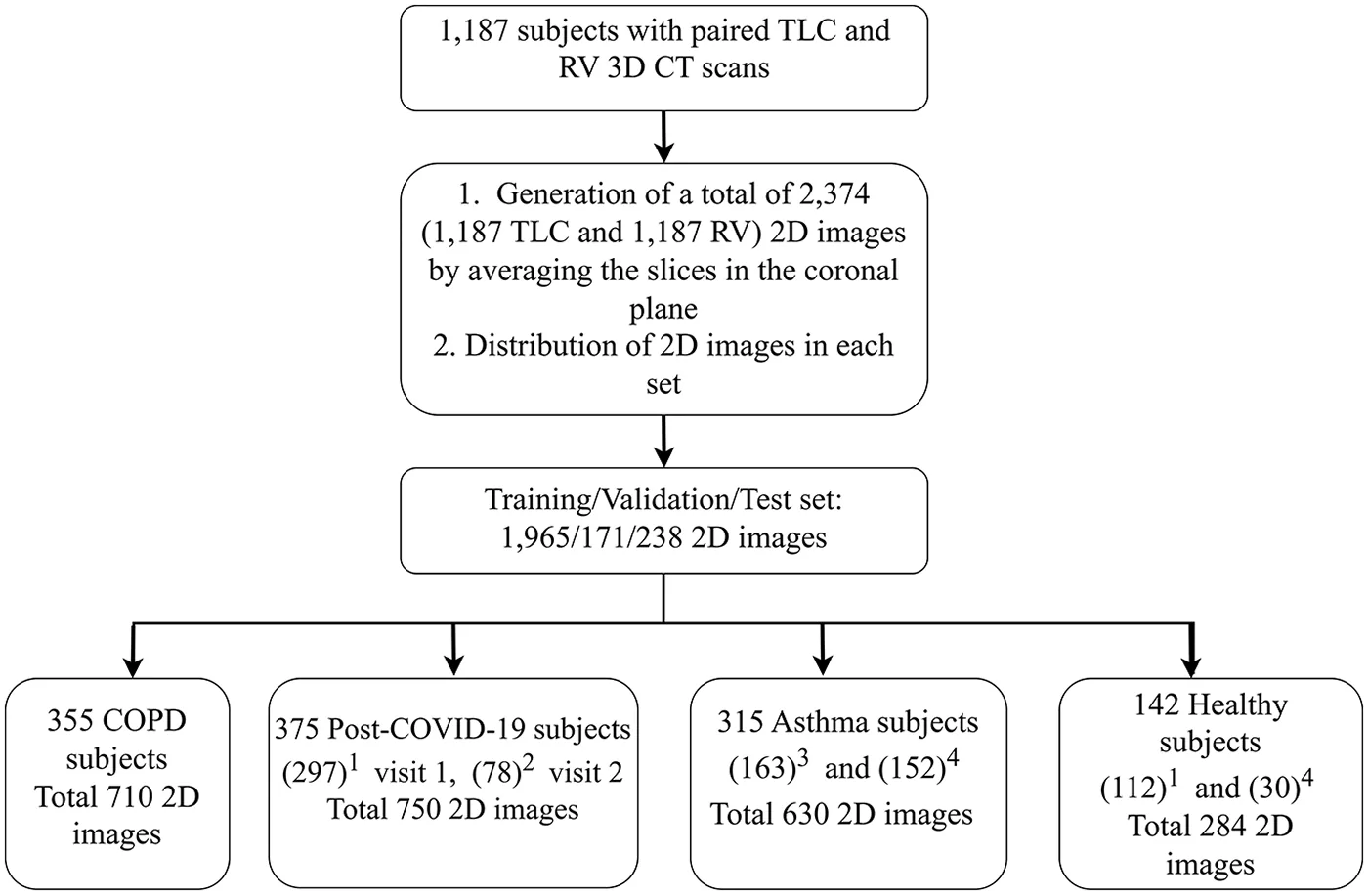
Dataset overview flowchart. From a cohort of 1,187 subjects (1,003 subjects with a single scan and 92 subjects with a follow-up scan after their initial visit) with paired three-dimensional (3D) computed tomography (CT) scans at total lung capacity (TLC) and residual volume (RV). A total of 2,374 2D images (mimicking chest x-rays) were generated for this study. The dataset comprises four cohorts. ^1^The post-COVID-19 subjects who tested positive for SARS-CoV-2 between February 2020 to January 2022, and 112 healthy subjects who were not infected with SARS-CoV-2 at the University of Iowa Hospitals & Clinics. The mean time interval between the diagnosis of COVID-19 and the first visit to the post-COVID-19 clinic was 172 days. ^2^Subjects approximately 3 years after the first visit. ^3^Subjects from the Severe Asthma Research Program (SARP) who underwent screening between February 2013 and November 2014, named SARP3. ^4^Subjects from SARP who underwent screening between July 2021 and August 2022, named SARP4. ^4^30 healthy subjects who were not infected from SARP4. Data from 92 follow-up subjects (33 asthma and 59 post-COVID-19) are used to assess disease improvement or progression. The average follow-up intervals were 2,865 days (∼8 years) for asthma subjects and 1,128 days (∼3 years) for post-COVID-19 subjects.

This retrospective study utilized de-identified data from multiple sources: (1) a post-COVID-19 cohort from the University of Iowa (UIOWA), (2) asthma subjects from the Severe Asthma Research Program (SARP) (23), (3) a COPD cohort from the Genetic Epidemiology of COPD (COPDGene) study collected at UIOWA (24), and (4) healthy controls from UIOWA and SARP (23). Figure 1 shows the dataset overview flowchart. All study protocols were approved by the respective institutional review boards. Inspiratory and expiratory chest CT images were acquired at TLC and RV. The post-COVID-19 cohort comprised 750 subjects, including 686 patients (91.5%) with post-COVID-19 only, 54 patients (7.2%) with pre-existing asthma, and 10 patients (1.3%) with pre-existing COPD. The 54 post-COVID-19 patients with pre-existing asthma and 10 with pre-existing COPD were not cross-contaminated between the training sets of the main asthma or COPD cohorts.

### 2.2 Image processing and qCT metrics

A total of 2,374 (1,187 TLC and 1,187 RV) 3D CT images were rescaled to a range of 0–1 for air-tissue to mitigate scanner variability and masked using VIDA Vision (VIDA Diagnostics, Coralville, Iowa) to segment lung regions. Structural and functional qCT metrics were then extracted from the TLC scans. Subsequently, the 3D CT images were compressed into 2D coronal average projections (mimicking chest x-rays) and SimpleITK (version 2.4.0) was used for further image processing. Structural metrics, including airway wall thickness (W_T_) and hydraulic diameter (D_h_) across lobes (LLL, RUL, RML, RLL, LUL; LLL = left lower lobe, RUL = right upper lobe, RML = right middle lobe, RLL = right lower lobe, LUL = left upper lobe) and specific branches such as the right main bronchus (RMB) and right intermediate bronchus (BronInt) at TLC were calculated. To reduce individual variability, normalized wall thickness (W*_T_) and hydraulic diameter (D*_h_), indicated by a superscripted asterisk, were computed using predicted values from healthy subjects matched for sex, age, and height (25).

Image registration was applied to the 3D CT volumes to derive functional metrics, including the determinant of the Jacobian matrix (hereafter referred to as Jacobian, ≥ 1), which measures local volume expansion from expiratory to inspiratory CT scans, and the anisotropic deformation index (ADI), where ADI=0 indicates perfectly isotropic deformation, while higher values reflect anisotropic deformation, meaning that the lung expands or contracts preferentially along specific directions (26). Structural metrics W*_T_ and D*_h_ were obtained from segmentation. In this study, we focused on major qCT metrics identified in previous work (27), including the Jacobian, AirT% (percent air trapping), D*_h, BronInt,_ W*_T, BronInt,_ W*_T, RUL_, W*_T, RML,_ D*_h, LLL_, ADI_LUL,_ and Jacobian_LUL_. Subscripts indicate specific lung regions, while metrics without subscripts represent total lung measurements.

### 2.3 Overall architecture

**Figure 2:**
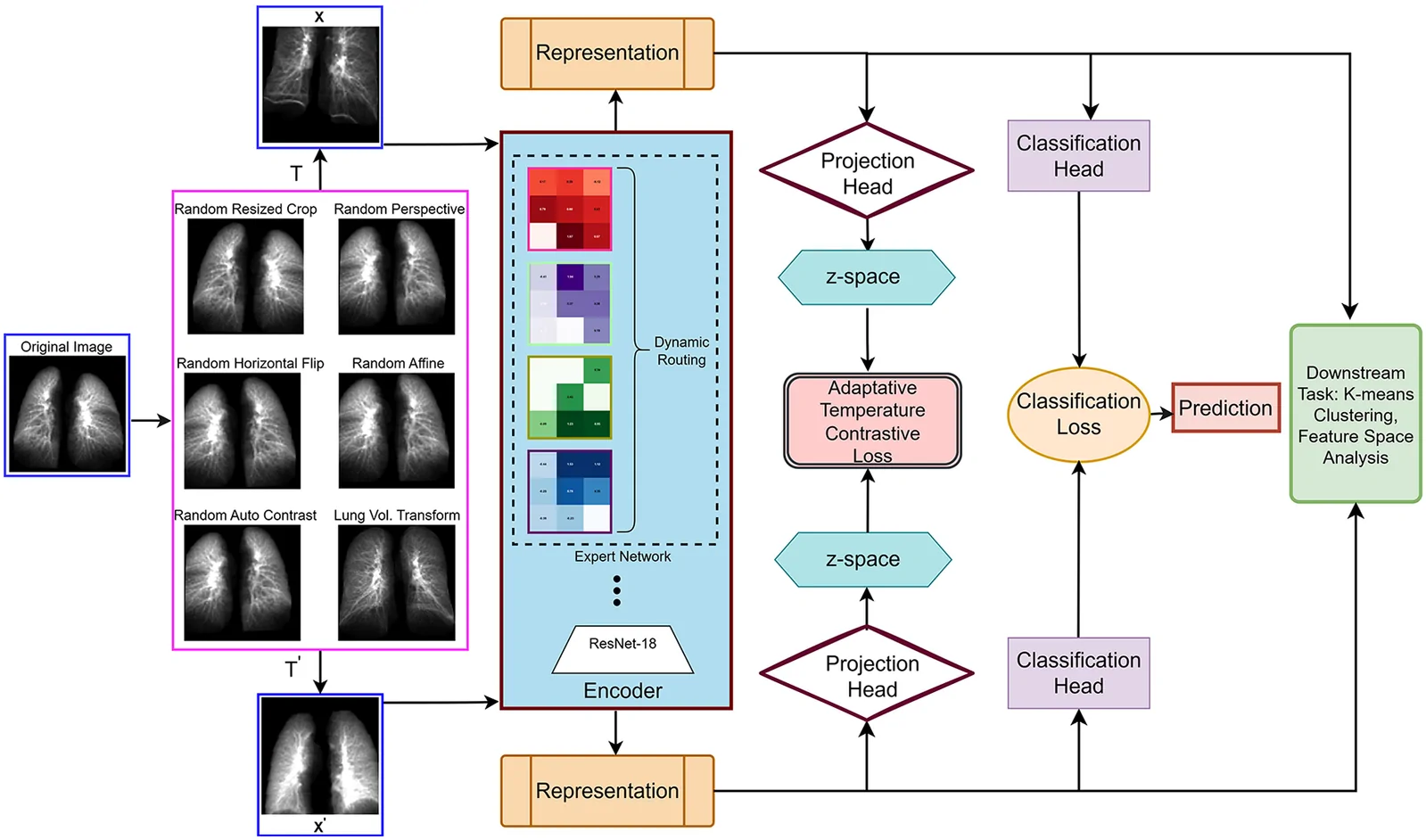
Overview of the proposed model workflow. The 3D CT images were normalized (0 for air, 1 for tissue), masked by lung segmentations, and averaged across the coronal plane to produce 2D projections, denoted by “Original Image,” that approximate chest x-rays. T and T ‘ denote two transformation types, and X and X ‘ are their corresponding transformed views. Each transformation is a composite of random resized cropping, random horizontal flipping, random perspective transformation, random affine transformation, random auto contrast, and a volume transform in which, with a certain probability, the original image under T is replaced by its counterpart at the opposite lung volume (i.e., TLC image is replaced by its corresponding RV image and vice versa). The probability for applying the volume transform was set to 0.4 in this study (11).

The overall model architecture is presented in Figure 2. Contrasting 2D image pairs are generated using two composite transforms, T and T′. In addition, a volume transform is applied with a probability 0.4, swapping an 2D image to its counterpart lung volume (TLC to RV and vice versa) to encourage volume-invariant feature learning (11).

### 2.4 Contrastive learning model

We adapted a ResNet-18-based contrastive learning framework and integrated expert-conditioned convolutions to enhance discriminative feature learning. Standard convolutional layers, which apply the same kernels throughout the entire image, may be insufficient to capture heterogeneity among different input classes. Motivated by the concept in (28), we proposed a conditionally parameterized convolutional layer that dynamically blends four expert convolutional kernels based on the input image.

#### 2.4.1 Expert-conditioned routing

Instead of using static convolutional kernels, the kernels are computed as a function of input image and parameterized as a linear combination of *n* convolutional kernels in Equation 1:

$$\begin{array}{c} W(x)=\overset{4}{\underset{n=1}{\sum }}\alpha_{n}(x)\cdot W_{n}\; \end{array}$$ (1)

where $W_{n}=\{W_{1},W_{2},W_{3},W_{4}\}\;$ are the same-sized expert convolutional kernels for four classes (three disease classes and one healthy class), and $\alpha_{n}(x)=\{\alpha_{1}(x),\alpha_{2}(x),\alpha_{3}(x),\alpha_{4}(x)\}$ are the corresponding scalar routing weights.

The routing weights are computed directly from the input image via a routing function that employs global average pooling (GAP), a linear layer, and a SoftMax activation (Equation 2).

$$\begin{array}{c} \alpha_{n}(x)=Softmax{\left(Linear(GAP(x))\right)}_{n}\;,\;n=1,2,3,4\; \end{array}$$ (2)

This routing computation is identical during training and at inference: it depends only on the input image *x,* and the class label is never supplied to this function at any stage. Ground-truth labels play no role in computing the routing weights directly. Instead, the expert kernels and the routing sub-network acquire class-discriminative behavior indirectly, through gradients backpropagation from the downstream classification loss during training.

The resulting routed kernel $W(x_{i})$ is used in place of a static kernel in the first convolutional layer of the ResNet-18 backbone, so that the encoder's output representation for each image is given by Equation 3.

$$\begin{array}{c} z_{i\;}=f_{\theta }(x_{i};W(x_{i})) \end{array}$$ (3)

Where *θ* denotes the backbone parameters excluding the expert kernels $W_{n}$.

#### 2.4.2 Encoder with projection and classification heads

The encoder mapped an input image (128 × 128 × 1) to a 512-dimensional representation $z_{i\;}$, which was then processed in parallel by two heads: a projection head, implemented as a multilayer perceptron for contrastive representation learning, and a classification head for class prediction. The representations were also used for k-means clustering and feature space analysis. The network contained a total of 11,254,025 trainable parameters.

#### 2.4.3 Adaptive temperature and training losses

An adaptive temperature scaling mechanism was employed, where the temperature parameter for the contrastive loss was dynamically adjusted based on the standard deviation of similarity scores within each training batch to optimize learning. The contrastive and total loss can be expressed as follows (13):

$$\begin{array}{c} \tau_{b}=\min(max(std(S),\;\tau_{min}),\;\tau_{max}),\;\;\;S=\{\;s_{i,j}\;:i,j\;\in B\} \end{array}$$ (4)

$$\begin{array}{c} s_{i,j}=\frac{{\left(z_{i}\right)}^{T}\cdot z_{j}}{∥z_{i}∥\cdot ∥z_{j}∥}\; \end{array}$$ (5)

$$\begin{array}{c} l(i,j)=-\log\frac{\exp(s_{i,j}/\tau_{b\;})\;}{\sum_{k=1}^{2N}1_{\left[k\;\neq i\right]}\cdot \exp(s_{i,k}/\tau_{b\;})\;}\; \end{array}$$ (6)

$$\begin{array}{c} L_{contrastive}=\frac{1}{2N}\overset{N}{\underset{k=1}{\sum }}l(2k-1,\;2k)+l(2k,\;2k-1) \end{array}$$ (7)

$$\begin{array}{c} L_{class}=-\frac{1}{2N}\overset{2N}{\underset{k=1}{\sum }}\overset{4}{\underset{c=1}{\sum }}y_{k,c}\log(p(y_{k,c})) \end{array}$$ (8)

$$\begin{array}{c} L_{total}=w_{1}L_{contrastive}+\;w_{2}L_{class} \end{array}$$ (9)

where $\tau_{b}$ is the adaptive temperature that is used in the contrastive loss for the current training batch *B* (Equation 4). $\tau_{min}$ is the lower bound on the temperature that is 0.05 and $\tau_{max}$ is the upper bound of value 1. $s_{i,j}$ is the cosine similarity matrix computed of samples *i* and *j* (Equation 5). l(i,j) represents the mechanism of a single-pair contrastive loss with batch-adaptive temperature mechanism $\tau_{b}$ as defined in Equation 6. The total loss ($L_{total})$ is the weighted average of batch contrastive loss ($L_{contrastive})$ in Equation 7 and the classification loss ($L_{class})$ in Equation 8. *c* is the ground-truth label that supervises the classification loss (Equation 8). *N* is the sample size of a mini batch (Equation 7 and Equation 8). $w_{1}$ and $w_{2}$ are weights for $L_{contrastive}$ and $L_{class}$, respectively in Equation 9.

#### 2.4.4 Inference pipeline

At inference, the model receives an unlabeled image *x* and executes the identical forward computation used during training (Equation 1–3), with the projection head and contrastive loss discarded. Only the routing sub-network, the ResNet-18 backbone, and the classification head are retained at inference time; the projection head is used solely during training and does not form the part of the inference pathway.

No class label is used at any stage of this computation. Since the routing function depends only on the input image during both training and inference, its behavior is identical across both phases, with no mechanism for label information to enter at inference time. Inference is therefore performed in a fully label-free manner.

### 2.5 Experimental settings

The proposed contrastive learning model was implemented in PyTorch (version 2.4.1). All experiments were conducted on an NVIDIA RTX A4500 GPU with 20 GB of VRAM. The model was trained using the Adam optimizer with an initial learning rate of 0.00025, weight decay of 0.05, and a batch size of 64 for 100 epochs. To benchmark performance, we compared our model against the baseline SimCLR (Simple framework for Contrastive Learning of visual Representations) (16).

### 2.6 Statistical analysis

Statistical analyses were performed in Python using SciPy (version1.14.1) and Pingouin (version 0.5.5). Welch's ANOVA was used for cluster comparisons, Kruskal–Wallis assessed statistical differences between post-COVID-19 subgroups with varying pre-existing conditions, and Wilcoxon signed-rank tests were applied for longitudinal analysis of follow-up patients between visits. In all analyses, *P* values < 0.05 were considered statistically significant.

## 3 Results

### 3.1 Subject characteristics

The demographic and pulmonary function test (PFT) characteristics of the four cohorts is summarized in Table 1. A total of 1,187 subjects were analyzed: healthy controls (*n* = 142), COPD (*n* = 355), post-COVID-19 (*n* = 375), and asthma (*n* = 315). Among the asthma subjects, 163 subjects were from SARP3 (screened February 2013 − November 2014) and 152 from SARP4 (screened between July 2021 − August 2022). In the post-COVID-19 cohort, 297 subjects were assessed at the initial visit (V1) and 78 were assessed at the follow-up visit (V2). A total of 33 asthma subjects and 59 post-COVID-19 subjects underwent follow-up scans. The average time intervals between visits were 2,865 days (∼8 years) for asthma subjects and 1,128 days (∼3 years) for post-COVID-19 subjects.

**Table 1:** Demographics and PFT data for all subjects.

Variable	Healthy	COPD	Post-COVID-19		Asthma	
			V1	V2	SARP3	SARP4
Age (y)	44.59 ± 14.28	68.87 ± 8.40	46.73 ± 15.32	47.12 ± 12.05	47.71 ± 14.38	53.74 ± 14.56
Height (cm)	170.99 ± 9.00	169.68 ± 9.46	169.72 ± 9.89	169.91 ± 9.02	166.14 ± 10.15	166.61 ± 19.67
BMI (kg/m^2^)	26.02 ± 4.20	30.10 ± 5.97	33.67 ± 9.53	32.25 ± 7.80	32.57 ± 7.94	31.62 ± 8.19
Sex						
Female	78 (54.92)	170 (48.15)	197 (66.10)	55 (70.51)	109 (66.87)	97 (63.8)
Male	64 (45.07)	183 (51.84)	101 (33.89)	23 (29.48)	54 (33.13)	55 (36.2)
Race: White/ African American/Other	120/5/17	351/2/0	—	68/2/8	103/38/22	107/27/18
Weight (kg)	76.39 ± 15.34	87.02 ± 19.75	96.93 ± 27.29	93.08 ± 22.94	89.68 ± 22.60	87.94 ± 22.59
Post FEV_1_% Predicted	101.05 ± 11.73	91.19 ± 19.99	94.43 ± 16.27	104.18 ± 14.28	81.04 ± 21.36	86.61 ± 19.99
Post FVC % Predicted	101.97 ± 11.55	93.08 ± 13.78	95.31 ± 16.86	104.27 ± 14.05	91.96 ± 17.73	98.14 ± 19.98
Post FEV_1_/FVC % Predicted	99.03 ± 6.16	72.88 ± 11.4	81.74 ± 7.80	80.64 ± 6.92	86.29 ± 15.03	86.97 ± 14.94

Data are means ± standard deviations, with percentages in parentheses. The racial category “Other” includes American Indian or Alaska Native, Asian, Native Hawaiian or other Pacific Islander. “—” indicates data not provided. The asthma class data are from the Severe Asthma Research Program (SARP), consisting of SARP3 (screened February 2013–November 2014) and SARP4 (screened July 2021–August 2022). The post-COVID-19 cohort includes 297 subjects at the initial visit (V1) and 78 subjects at the follow-up visit (V2), acquired approximately 3 years after V1.

### 3.2 Model performance

**Figure 3:**
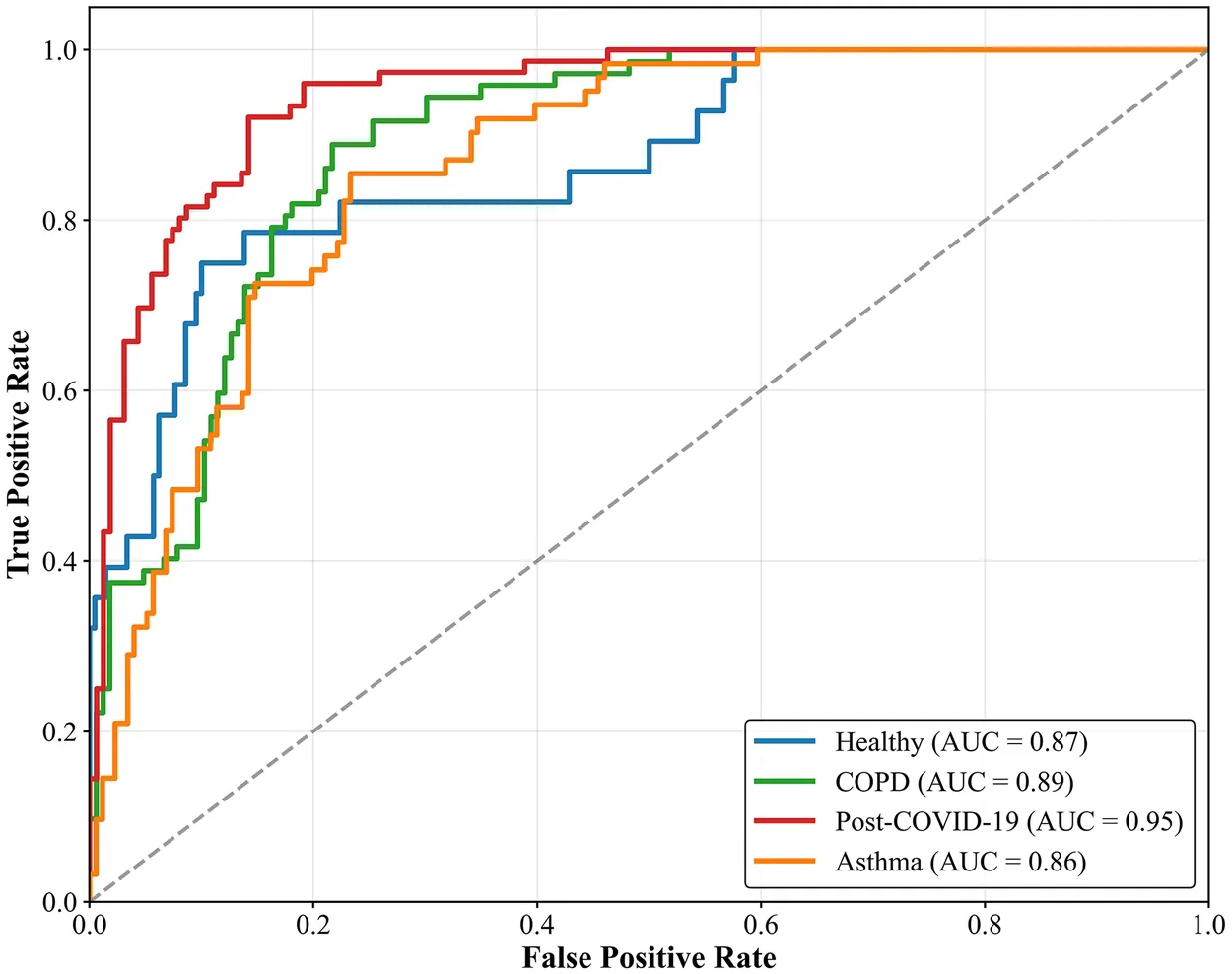
Receiver operating characteristic (ROC) curve and area under the curve (AUC) score of the model on the test set.

**Figure 4:**
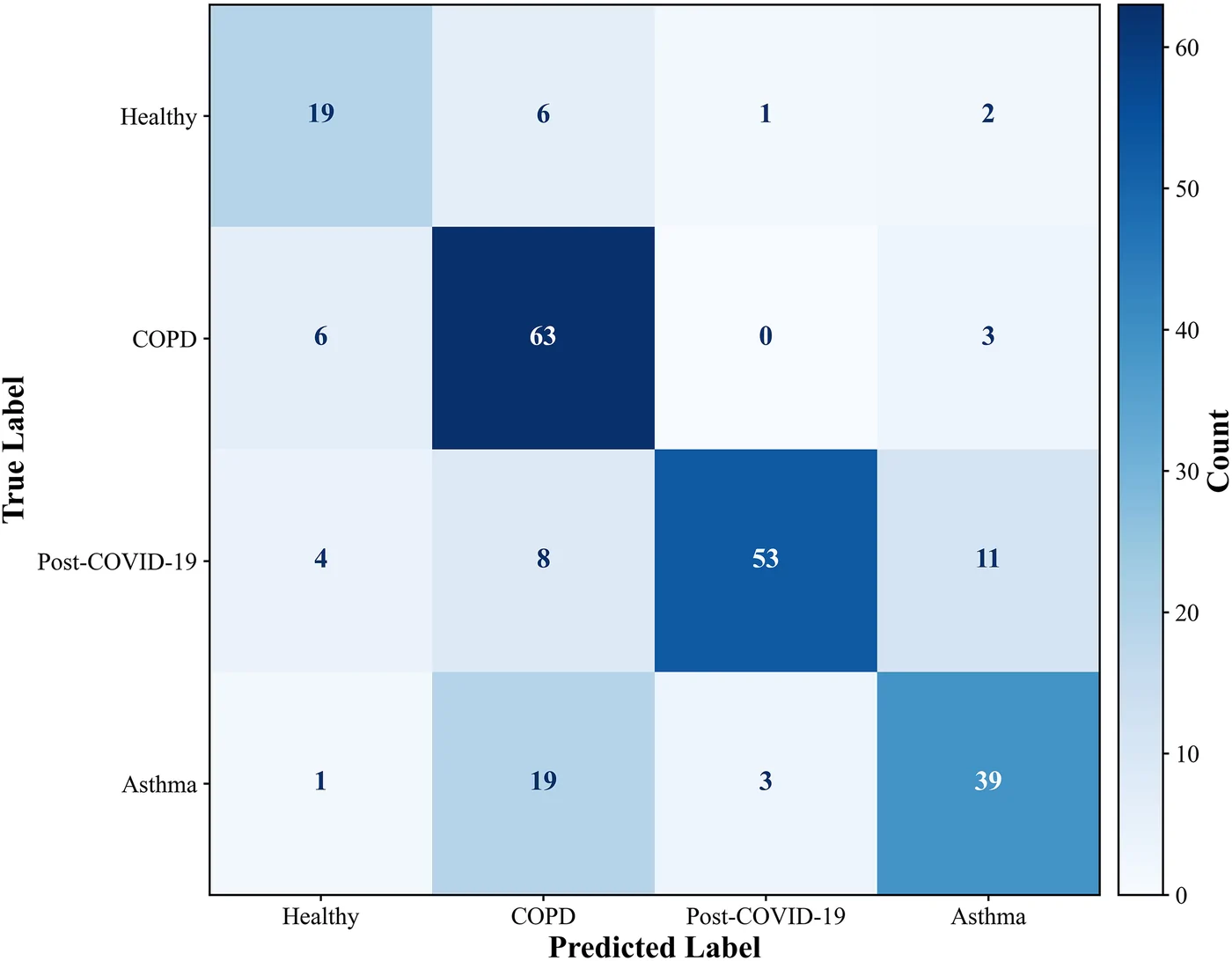
Confusion matrix of the proposed model.

The proposed contrastive learning model achieved a macro-AUC of 89.3% and an overall balanced accuracy of 73.1% on the internal test set in distinguishing the four classes. Figure 3 shows the model performance. The confusion matrix (Figure 4) illustrates class-wise prediction results. The model showed high accuracy for the COPD class, correctly identifying 63 cases, with 6 and 3 misclassified as healthy and asthma, respectively, demonstrating its ability to capture distinctive COPD features. For the post-COVID-19 class, 53 cases were correctly classified, with 11 misclassified as asthma and 8 as COPD. The healthy class had 19 correct predictions, with 6 misclassified as COPD. Asthma exhibited the greatest confusion, with 39 correctly classified cases and 19 misclassified as COPD, reflecting overlapping imaging characteristics between the two diseases.

 Table 2 summarizes the performance comparison between the proposed model and the baseline SimCLR. The proposed model achieved an F1-score of 0.717, outperforming SimCLR (0.637). Class-wise AUC analysis further highlights the improvement, with the post-COVID-19 class reaching 0.95 for the proposed model compared to 0.90 for SimCLR.

**Table 2:** Performance comparison between the baseline model and the proposed model.

Variable	Healthy	COPD	Post-COVID-19	Asthma	Mean
Sensitivity % (TP/TP + FN)					
SimCLR	67.9 (19/28)	77.8 (56/72)	65.8 (50/76)	50.0 (31/62)	65.4
Proposed	67.9 (19/28)	87.5 (63/72)	69.7 (53/76)	62.9 (39/62)	72.0
Specificity % (TN/ T*N* + FP)					
SimCLR	89.5 (188/210)	79.5 (132/166)	92.6 (150/162)	92.0 (162/176)	88.4
Proposed	94.8 (199/210)	80.1 (133/166)	97.5 (158/162)	90.9 (160/176)	90.8
Precision % (TP/TP + FP)					
SimCLR	46.3 (19/41)	62.2 (56/90)	80.6 (50/62)	68.9 (31/45)	64.5
Proposed	63.3 (19/30)	65.6 (63/96)	93.0 (53/57)	70.9 (39/55)	73.2
F1-score					
SimCLR	0.551	0.691	0.725	0.579	0.637
Proposed	0.655	0.750	0.797	0.667	0.717
AUC					
SimCLR	0.85	0.85	0.90	0.85	0.862
Proposed	0.87	0.89	0.95	0.86	0.893
Support (TP + FN)	28	72	76	62	238

TP, True Positive; FP, False Positive; TN, True Negative; FN, False Negative.

### 3.3 Feature embeddings

**Figure 5:**
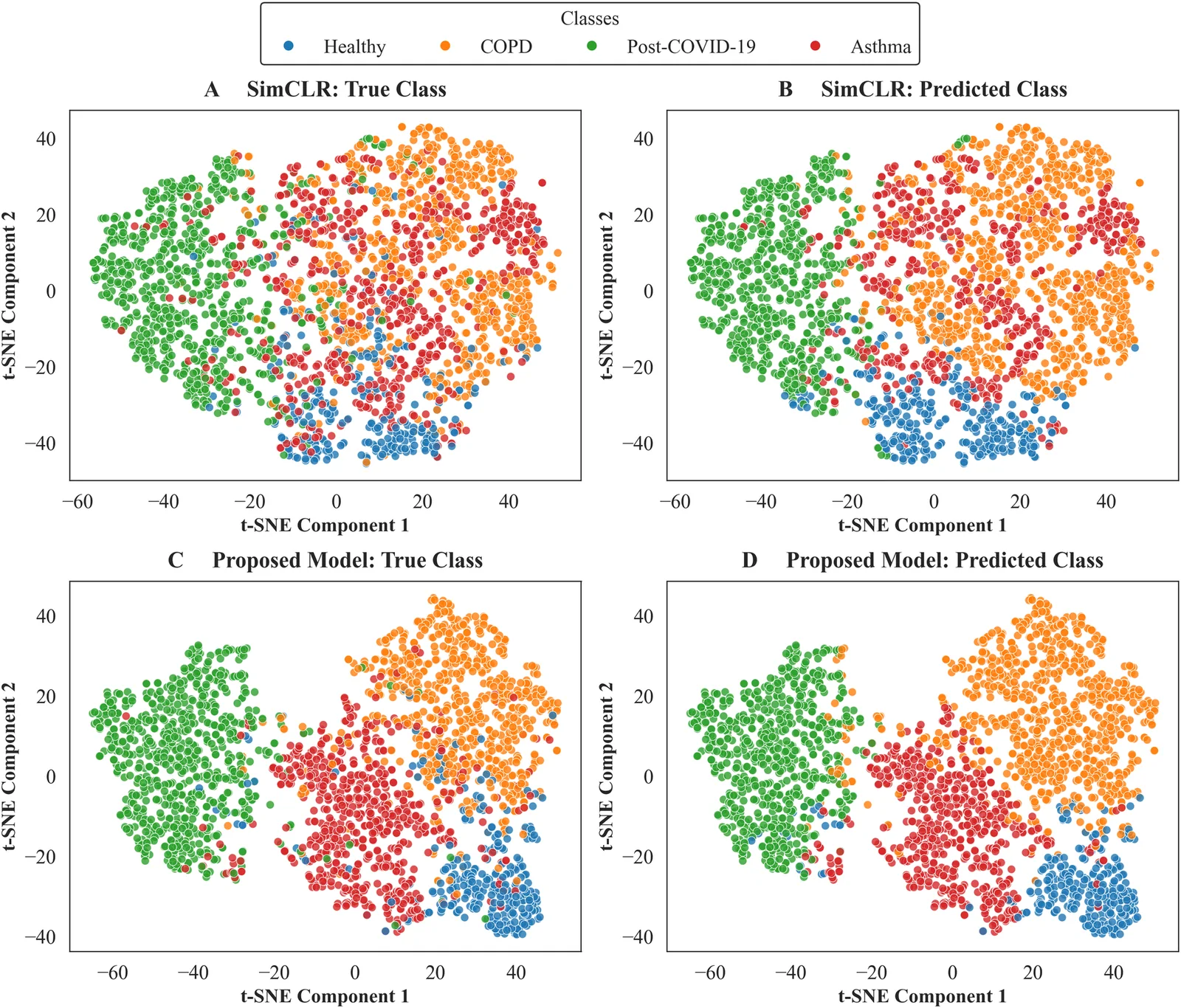
t-SNE embedding visualization of the learned feature space for the SimCLR (16) model: **(A)** true class and **(B)** predicted class, and for the proposed model: **(C)** true class and **(D)** predicted class. The SimCLR model shows substantial overlap between diseases, indicating poor separability of disease-specific representations, whereas the proposed model demonstrates more discriminative features.

**Figure 6:**
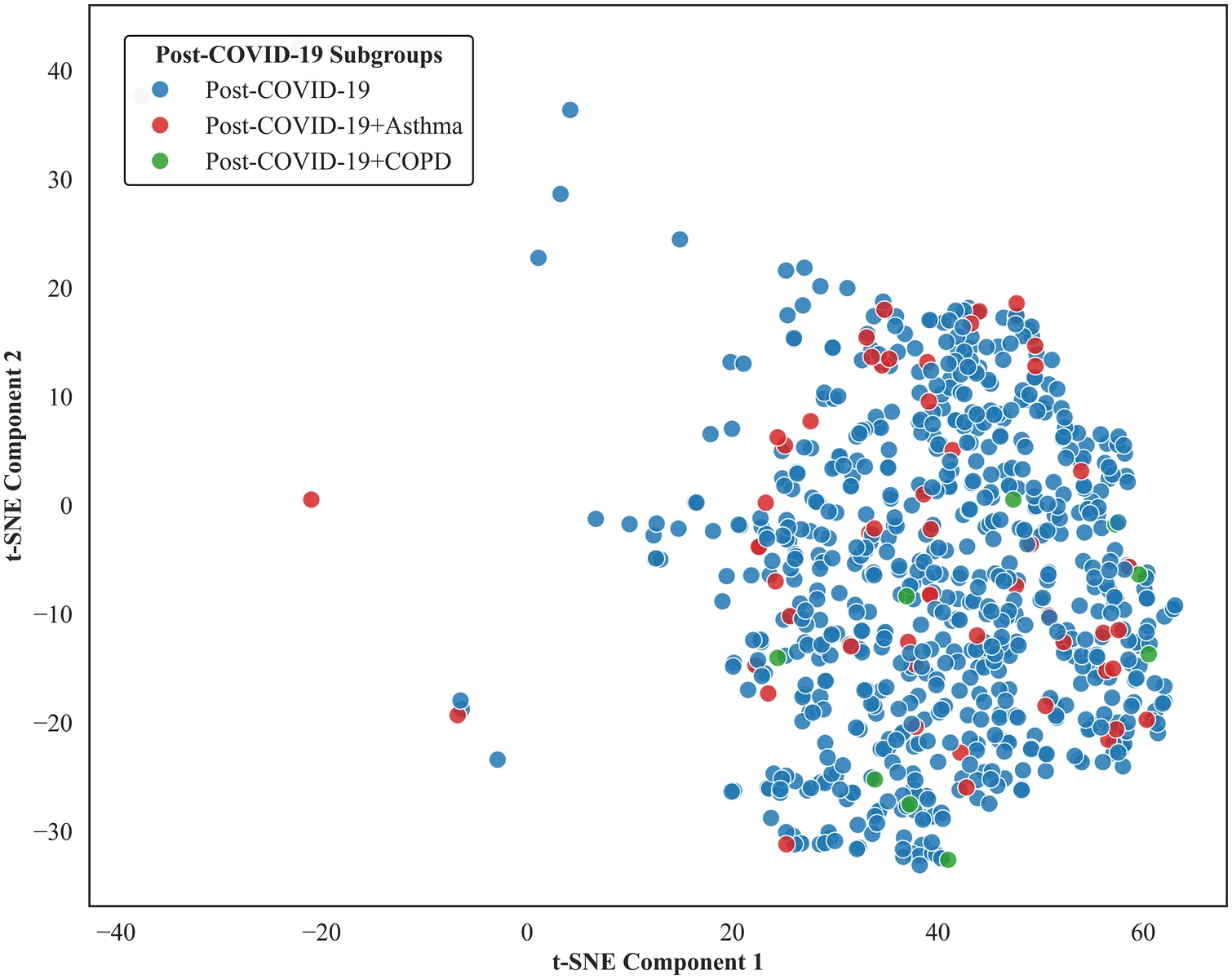
t-SNE embedding visualization for post-COVID-19 subgroups only.

To evaluate feature separability, the learned representations were projected into two dimensions using t-SNE. As shown in Figure 5, the proposed model produced more discriminative embeddings that better preserve disease-specific distinctions. Notably, post-COVID-19 cases were well separated from asthma and COPD. To assess potential confounding effects from pre-existing conditions, the post-COVID-19 cohort was split into post-COVID-19 only (*n* = 686), post-COVID-19 with asthma (*n* = 54), and post-COVID-19 with COPD (*n* = 10) subgroups. Their distributions within the t-SNE embedding space showed substantial overlap, with no statistically significant differences observed (Figure 6; Kruskal–Wallis test, *P* > 0.05 for all feature comparisons). Therefore, the post-COVID-19 subjects form a distinct cluster, irrespective of the presence or absence of pre-existing asthma or COPD (29).

**Figure 7:**
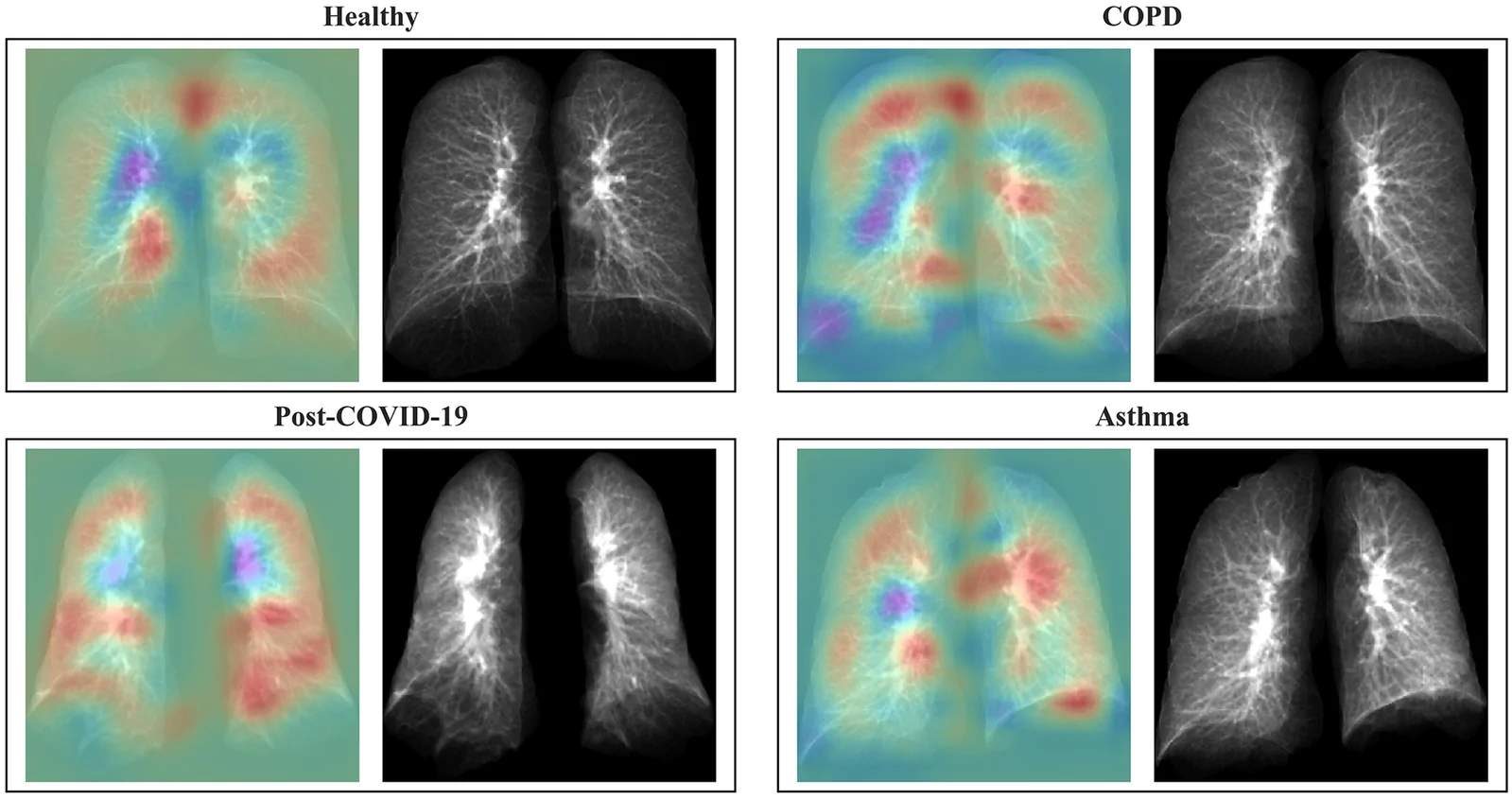
Gradient-weighted class activation mapping (grad-CAM) for representative subjects from each cohort. Each pair shows the Grad-CAM overlay (left) and the corresponding original 2D projection (right). The activation maps show the important regions for determining whether subjects belong to the Healthy, COPD, Post-COVID-19, or Asthma cohort, with red indicating regions of highest model activation and violet indicating regions of lowest activation.

To spatially validate the model's decision-making, gradient-weighted class activation mapping (Grad-CAM) were generated for a representative subject from each cohort (Figure 7). The activation maps illustrate that the model focuses on disease-specific lung regions, with spatially distinct patterns observed across cohorts.

### 3.4 Characteristics of subject clusters

Unsupervised k-means clustering was applied to the concatenated TLC and RV latent representations to independently assess whether the learned embeddings capture distinct, discriminative features across the four classes. Silhouette analysis identified four as the optimal number of clusters, yielding a clustering accuracy of 88.5%. The four clusters (C0–C3) were characterized as follows: C0 (*n* = 214, 61.21% healthy), C1 (*n* = 334, 91.92% COPD), C2 (*n* = 379, 96.83% post-COVID-19), and C3 (*n* = 260, 94.23% asthma). The lower accuracy of the healthy cluster (C0) is attributable to overlapping imaging characteristics with asthma and COPD. Detailed subject distributions and related data are provided in the Supplementary material.

The four identified clusters exhibited distinct qCT profiles (Table 3). C2 (post-COVID-19) had the thickest airway walls (W*_T, BronInt_: 0.81; W*_T, RUL_: 0.71; W*_T, RML_: 0.64). Air trapping was elevated in C3 (asthma) and C1 (COPD) (AirT%: 9.93% and 9.80%, respectively), while lung deformability was highest in C0 (healthy controls; Jacobian: 2.70).

**Table 3:** Comparison of qCT metrics across disease-dominant clusters.

Variable	Wilk λ Value	Healthy (C0)	COPD (C1)	Post-COVID-19 (C2)	Asthma (C3)	*P* Value*
W*_T, RML_	0.678	0.53 ± 0.05	0.55 ± 0.05	0.64 ± 0.06	0.58 ± 0.05	<.001***
Jacobian	0.537	2.70 ± 0.61	2.11 ± 0.33	2.09 ± 0.56	1.94 ± 0.41	<.001***
W*_T, BronInt_	0.490	0.72 ± 0.09	0.72 ± 0.08	0.81 ± 0.07	0.73 ± 0.06	<.001***
ADI_LUL_	0.452	0.69 ± 0.15	0.66 ± 0.16	0.53 ± 0.19	0.55 ± 0.19	<.001***
AirT%	0.423	3.04 ± 4.00	9.80 ± 9.43	2.59 ± 4.06	9.93 ± 9.72	<.001***
W*_T, RUL_	0.402	0.61 ± 0.09	0.62 ± 0.11	0.71 ± 0.09	0.62 ± 0.10	<.001***
D*_h, BronInt_	0.397	0.66 ± 0.07	0.66 ± 0.06	0.67 ± 0.06	0.68 ± 0.06	<.05*
D*_h, LLL_	0.396	0.35 ± 0.06	0.34 ± 0.06	0.34 ± 0.05	0.34 ± 0.06	<.01**
Jacobian_LUL_	0.395	2.27 ± 0.51	1.82 ± 0.26	1.81 ± 0.45	1.68 ± 0.33	<.001***

Data are presented as means ± standard deviations. C0, C1, C2, and C3 refer to cluster 0, cluster 1, cluster 2, and cluster 3, respectively.

Wilk λ Value represents a stepwise forward variable selection method (36).

ADI, Anisotropic Deformation Index; D*_h_, Normalized Hydraulic luminal Diameter; AirT%, Percent air trapping; LLL, Left Lower Lobe; LUL, Left Upper Lobe; RML, Right Middle Lobe; RUL, Right Upper Lobe; RMB, Right Main Bronchus; BronInt, Right Intermediate Bronchus; W*_T_, Normalized Wall Thickness (25).

Subscripts indicate lung regions, while variables without subscripts represent whole-lung measurements.

* , **, and *** indicate levels of statistical significance.

### 3.5 Feature space analysis in follow-up patients

**Figure 8:**
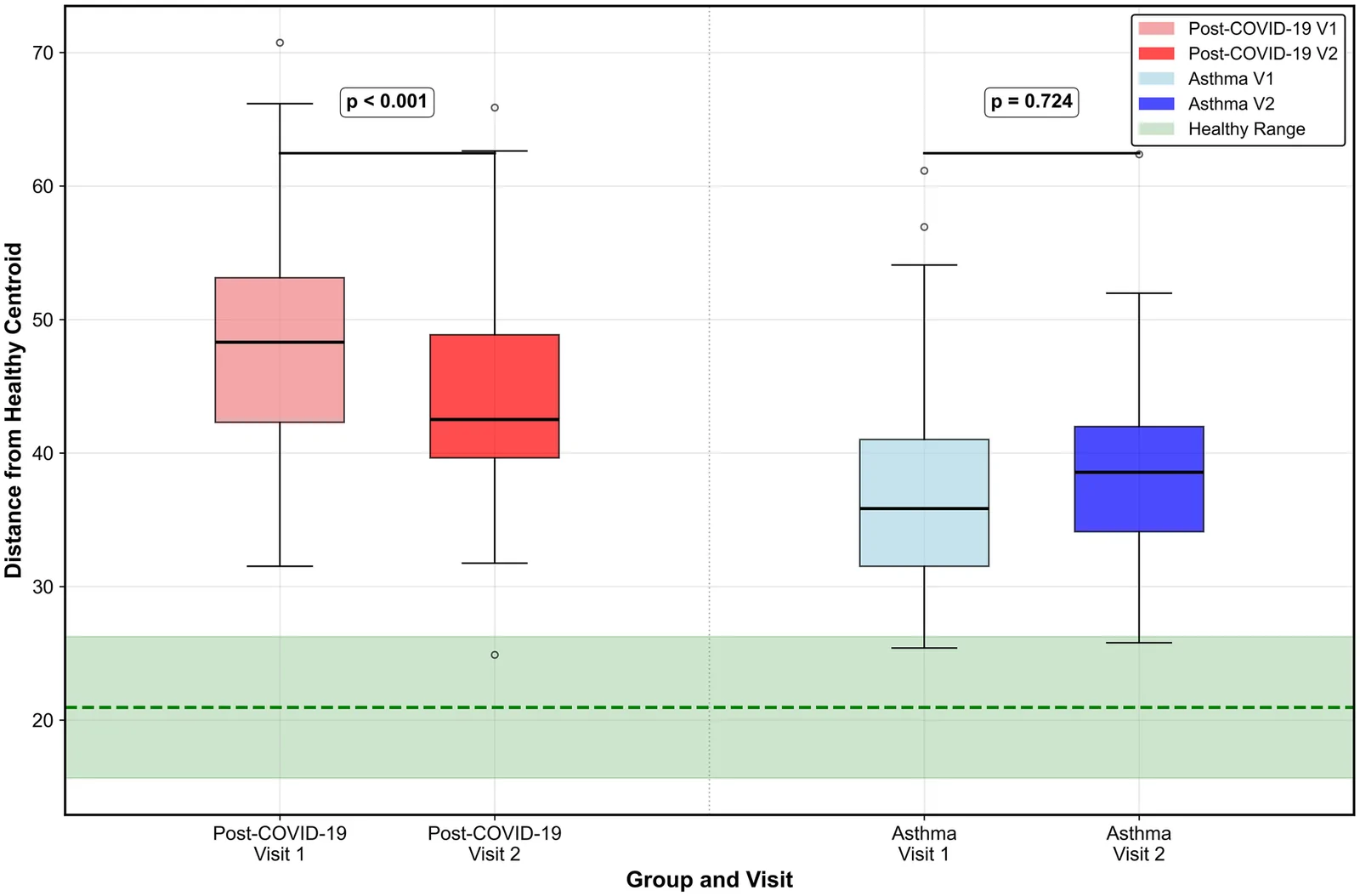
Quantitative longitudinal analysis of disease using the derived feature space distances. Box plots display the distribution of Euclidean distances from a healthy reference centroid for post-COVID-19 (red) and asthma (blue) cohorts at two clinical time points denoted by V1 (initial visit) and V2 (follow-up visit). The green shaded region represents the healthy population reference range (mea*n* ± one standard deviation). Statistical significance between visits was determined using Wilcoxon signed-rank tests. Post-COVID-19 patients demonstrated significant improvement toward the healthy state (*P* < 0.001), while asthma patients showed stable disease state (*P* = 0.724).

The embeddings from two visits for post-COVID-19 (*n* = 59) and asthma (*n* = 33) patients were analyzed to assess longitudinal changes. As shown in Figure 8, the disease progression or improvement was defined by the change in Euclidean distance between each patient's disease center and that of the healthy controls in the t-SNE feature space from V1 to V2—greater distance indicated disease progression, whereas shorter distance indicated disease improvement.

Post-COVID-19 subjects exhibited a statistically significant decrease in distance, indicating disease improvement over the approximately three-year follow-up period. These observations are consistent with PFT data, which demonstrated statistically significant improvements in both post FEV_1_% Predicted (98.40 ± 13.60 to 104.02 ± 14.16; *P* < 0.001) and post FVC % Predicted (98.96 ± 14.25 to 105.06 ± 14.10; *P* < 0.001). In contrast, asthma subjects showed a non-significant increase in distance over roughly eight years, suggesting stable disease state with minimal disease progression. This longitudinal stability was also mirrored in the clinical spirometry, where no significant differences were observed between visits for post FEV_1_% Predicted (82.30 ± 23.04 to 84.88 ± 23.53; *P* > 0.05) or post FVC % Predicted (95.65 ± 17.72 to 97.48 ± 19.18; *P* > 0.05).

### 3.6 Learned embeddings capacity to predict qCT metrics

A neural network regressor was trained on the learned embeddings to predict key qCT metrics. The embeddings showed strong predictive capacity for the global feature Jacobian ($R^{2}=0.61)$ and the lobar feature Jacobian_LUL_ ($R^{2}=0.57$). Performance varied across other metrics, with moderate success for ADI_LUL_ ($R^{2}=0.16)$, $W_{T,\;\mathrm{BronInt}}^{*}$ ($R^{2}=0.11)$, $W_{T,\;\mathrm{RML}}^{*}$ ($R^{2}=0.08)$, and $D_{h,\;\mathrm{LLL}}^{*}$ ($R^{2}=0.10)$. Predictive performance for AirT% was negligible.

## 4 Discussion

Building on our previous contrastive learning work on post-COVID-19 subjects using 2D CXR-like images (11), we developed an expert-conditioned contrastive learning framework that incorporated adaptive temperature scaling and lung volume transformation to enable robust, volume-invariant feature learning for multiclass respiratory disease classification.

The model's effectiveness was demonstrated through several key findings. First, visualization of the learned embeddings revealed clear class separation, especially highlighting post-COVID-19 as a distinct class. Second, the embeddings from two visits reflected disease improvement or progression across patient follow-up subgroups. Third, the model was computationally efficient: each epoch took approximately 2.6 min, with each batch processed within a second, resulting in a total training time of 3.74 h. This efficiency suggests that the approach could be adapted for clinical use without substantial computational burden. Although projecting 3D CT data into 2D CXR-like images inevitably results in some loss of spatial information, this framework has the potential to bridge conventional CXR imaging with the rich disease cluster-specific phenotypes derived from CT using either handcrafted qCT metrics (27, 29–33) or deep-learning-based imaging biomarkers (34). The learned embeddings serve not only as discriminative features for disease classification but also as representations that can be linked to CT-derived cluster-specific phenotypic characteristics, thereby enabling clinically interpretable characterization of lung disease from widely available CXR imaging.

The unsupervised k-means clustering served as an internal validation: if the contrastive learning model learned clinically meaningful representations, k-means should recover clusters aligned with the known disease groups without using labels. While we expected four clusters, deviations could be informative. Fewer clusters (e.g., three) would suggest overlapping imaging features between disease groups, whereas more clusters (e.g., five) might indicate subtypes or heterogeneous phenotypes within our cohorts. The independent recovery of four clusters confirms that the embeddings captured disease-specific patterns. The lower accuracy in the healthy cluster likely reflects shared imaging features with disease cases, near-normal features in some disease subjects, or features arising from aging-related abnormality in healthy individuals.

The misclassification between asthma and COPD shown in Figure 4 is not uncommon, as these conditions share disease phenotype overlaps, including airflow obstruction and structural changes (35). This diagnostic challenge persists even in clinical practice and is not unique to image-based models. Our clustering analysis further supports this overlap, as asthma and COPD demonstrated similar discriminative qCT biomarker profiles (Table 3), confirming that these conditions occupy overlapping regions in the learned feature space. However, integrating additional clinical features such as disease history may improve the model's performance.

Importantly, the model's sensitivity extended beyond static classification, enabling detection of disease development. The embeddings captured clinically relevant changes across patient follow-up subgroups, indicating that they encoded dynamic disease characteristics rather than merely static patterns. This sensitivity to temporal changes further indicates the clinical relevance of the embeddings and highlights their potential utility for monitoring disease trajectories and treatment responses.

We further evaluated whether deep-learning-based embeddings—features without explicit pathophysiological meaning—could predict qCT metrics, which serve as feature-engineered biomarkers with known pathophysiological interpretations. The embeddings strongly predicted lung volumetric expansion (Jacobian and Jacobian_LUL_) and moderately predicted anisotropy deformation (ADI_LUL_), airway wall thickness ($W_{T,\;\mathrm{BronInt}}^{*}$ and $W_{T,\;\mathrm{RML}}^{*}$), and airway diameter ($D_{h,\;\mathrm{LLL}}^{*}$), all key qCT metrics used to differentiate disease-dominant clusters (Table 3). These findings suggest that the model's representations encode meaningful information about lung function and biomechanics.

In addition to the information loss associated with the 3D-to-2D dimensional reduction, the limited predictive performance for AirT% may also be attributable to the composition of the study cohort. Specifically, the COPD cohort in the present study had a mean AirT% of 9.80 ± 9.43. In comparison, the current-smoker COPD cluster 1 (predominantly GOLD 0) had a mean AirT% of approximately 7.4%, whereas the former-smoker COPD cluster 1 (predominantly GOLD 0–1) had a mean AirT% of approximately 14.5% (30, 31). Because our cohort consisted primarily of subjects with mild COPD or those at risk for COPD, the range of AirT% values was relatively limited, reducing the regression model's ability to learn a robust mapping for this metric. Including subjects with more advanced COPD (e.g., the former-smoker cluster 4, predominantly GOLD 3–4, with a mean AirT% of approximately 62%) may broaden the range of AirT% values and improve the model's predictive performance for AirT%.

Our work advances the field by addressing multiclass disease classification, in contrast to prior studies that focused on binary tasks (e.g., asthma vs. healthy) or subtyping within a single disease. The expert-conditioned routing network and adaptive temperature scaling enable the model to prioritize clinically relevant features, producing embeddings that capture meaningful pathophysiological distinctions and achieve superior interpretability and performance compared to generic contrastive learning SimCLR.

This study had several limitations. First, the sample size for the healthy class was smaller than the three disease cohorts, representing a moderate class imbalance, and no explicit oversampling, under sampling, or class-weighted loss was applied in the present study. To reduce the influence of unequal class sizes during model evaluation, macro-averaged AUC and balanced accuracy were reported, both of which give equal consideration to each class rather than allowing the larger disease groups to dominate the overall performance estimate. However, these evaluation metrics do not eliminate the possibility that class imbalance affected representation learning during training. Future studies could evaluate class-weighted classification or contrastive losses, balanced batch sampling, or targeted augmentation and oversampling of the healthy class to improve minority-class discrimination; these approaches should be assessed using independent validation cohorts. Second, although the model processes 2D images—which does not fully exploit 3D CT information and may limit feature characterization—this design facilitates future transfer learning to chest x-rays, enhancing accessibility and broadening clinical applicability. Third, the regression model demonstrated limited ability to predict AirT%, possibly due to the disease severity distribution of the COPD cohort, as AirT% accumulates predominantly in advanced-stage disease with substantial values observed only in GOLD 3–4 dominant imaging clusters (30, 31). Fourth, this model will need to be tested on prospective, independent cohorts from different institutions to establish generalizability. Fifth, the model was not explicitly evaluated for age-related bias, and age was not incorporated as an input variable or balancing criterion during training. Because age differed substantially across cohorts, particularly between the COPD and healthy groups, some learned features may reflect age-associated structural changes in addition to disease-specific imaging characteristics. Future work should include age-matched or age-stratified analyses, evaluation of performance across age subgroups, and statistical assessment of associations between learned embeddings and age. Where sufficiently large datasets are available, age-adversarial or confounder-adjusted representation-learning strategies could also be considered.

In conclusion, an expert-conditioned contrastive learning framework demonstrated that the learned latent representations effectively distinguish classes, particularly differentiating the post-COVID-19 class from others, and sensitively detect disease improvement or progression. The model's high AUC and clear separation in embedding visualizations validate the discriminative power of these embeddings. The independent recovery of four clusters through unsupervised k-means analysis further confirms that the embeddings encode disease-specific information. This approach not only enhances diagnostic accuracy but also provides interpretable insights into disease phenotypes. By bridging deep learning with clinical expertise, it paves the way for more precise and equitable diagnosis of complex respiratory conditions. External validation on prospective, independent cohorts from different institutions, and further investigation of the embeddings captured by the model, are needed to establish generalizability and clinical utility.

## Data Availability

The datasets presented in this article are not readily available Due to privacy restrictions, the data analyzed in this study include human chest CT scans obtained from multiple sources. Requests for access to University of Iowa (UIOWA) data may be directed to Professor Alejandro Comellas (Department of Internal Medicine, University of Iowa, alejandro- comellas@uiowa.edu). Access to Severe Asthma Research Program (SARP) data is governed by the SARP data access committee and must be requested through established SARP data-sharing procedures at http://www.severeasthma.org/.
