# Supplementary material for: Discriminative feature learning for multiclass lung disease classification using contrastive learning
Source: Front Radiol. 2026 Aug 19;6:1824991. doi: 10.3389/fradi.2026.1824991 (PMC13536901; doi:10.3389/fradi.2026.1824991)
Supplement: Supplementary file 1 [file Table1.docx]

Supplementary Material

# 1 Supplementary Tables

**Supplementary Table 1** Distribution of subjects in each cluster.

| Class | Healthy | COPD | Post-COVID-19 | Asthma |
| --- | --- | --- | --- | --- |
| Cluster 0 | 131 | 35 | 2 | 46 |
| Cluster 1 | 7 | 307 | 3 | 17 |
| Cluster 2 | 3 | 2 | 367 | 7 |
| Cluster 3 | 1 | 11 | 3 | 245 |

**Supplementary Table 2** Distribution of subject’s sex by cluster.

| Cluster | Male | Female |
| --- | --- | --- |
| 0 | 82 (38.3%) | 132 (61.7%) |
| 1 | 177 (53.3%) | 155 (46.7%) |
| 2 | 126 (33.2%) | 253 (66.8%) |
| 3 | 95 (37%) | 162 (63%) |
